# Role of miR‐218‐GREM1 axis in epithelial‐mesenchymal transition of oral squamous cell carcinoma: An in vivo and vitro study based on microarray data

**DOI:** 10.1111/jcmm.15972

**Published:** 2020-10-27

**Authors:** Yanpeng Wang, Yifeng Jiang, Long Chen

**Affiliations:** ^1^ Department of E.N.T. Linyi People's Hospital Linyi China; ^2^ Department of Stomatology Shandong Medical College Linyi China; ^3^ Department of Stomatology Linyi People's Hospital Linyi China

**Keywords:** epithelial‐mesenchymal transition, Gremlin1 gene, liver metastases nodes, microRNA‐218, oral squamous cell carcinoma, TGF‐β signalling pathway

## Abstract

Oral squamous cell carcinoma (OSCC) is a prevalent cancer that develops in the head and neck area and has high annual mortality despite optimal treatment. microRNA‐218 (miR‐218) is a tumour inhibiting non‐coding RNA that has been reported to suppress the cell proliferation and invasion in various cancers. Thus, our study aims to determine the mechanism underlying the inhibitory role of miR‐218 in OSCC. We conducted a bioinformatics analysis to screen differentially expressed genes in OSCC and their potential upstream miRNAs. After collection of surgical OSCC tissues, we detected GREM1 expression by immunohistochemistry, RT‐qPCR and Western blot analysis, and miR‐218 expression by RT‐qPCR. The target relationship between miR‐218 and GREM1 was assessed by dual‐luciferase reporter gene assay. After loss‐ and gain‐of‐function experiments, OSCC cell proliferation, migration and invasion were determined by MTT assay, scratch test and Transwell assay, respectively. Expression of TGF‐β1, Smad4, p21, E‐cadherin, Vimentin and Snail was measured by RT‐qPCR and Western blot analysis. Finally, effects of miR‐218 and GREM1 on tumour formation and liver metastasis were evaluated in xenograft tumour**‐**bearing nude mice. GREM1 was up‐regulated, and miR‐218 was down‐regulated in OSCC tissues, and GREM1 was confirmed to be the target gene of miR‐218. Furthermore, after up‐regulating miR‐218 or silencing GREM1, OSCC cell proliferation, migration and invasion were reduced. In addition, expression of TGF‐β signalling pathway‐related genes was diminished by overexpressing miR‐218 or down‐regulating GREM1. Finally, up‐regulated miR‐218 or down‐regulated GREM1 reduced tumour growth and liver metastasis in vivo. Taken together, our findings suggest that the overexpression of miR‐218 may inhibit OSCC progression by inactivating the GREM1‐dependent TGF‐β signalling pathway.

## 1 INTRODUCTION

Squamous cell carcinoma is the most prevalent cancer of the head and neck, with oral squamous cell carcinoma (OSCC) accounting for 90% of the cases.^1^ OSCC is the eight most common cancer in the USA, with over 48 000 new OSCC cases diagnosed annually.^2^ The survival rate for OSCC patients is only about 50%.^3^ Despite expenditure of significant efforts in improving the diagnostic and therapeutic approaches of OSCC, the mortality rate in OSCC patients remains unchanged in recent years.^4^ Moreover, OSCC patients will often suffer from functional and cosmetic defects following treatment.^5^ Lifestyle, environment and genetic background are important factors associated with the development of OSCC,^6^ and the main events related to OSCC‐induced mortality are metastasis and recurrence. Therefore, it is important to identify biological factors and regulatory molecular pathways that might lead to improved treatment for OSCC.^7^

The abnormal expression of microRNAs (miRNAs) can serve as tumour or oncogene suppressors, capable of regulating various biological processes such as development, differentiation, apoptosis and cell proliferation.^8^ Multiple types of cancer, including OSCC, have been observed to have dysregulated expression of miRNAs.^7^ miR‐218, a vertebrate‐specific intronic miRNA that is expressed along with its host gene tumour suppressor gene SLIT2/3 plays a tumour suppressive role by targeting numerous oncogenes associated with cell proliferation, apoptosis and invasion.^9^ According to prediction results obtained from Bioinformatic websites, Gremlin1 (GREM1) could be a target gene of miR‐218, which led us to hypothesize that miR‐218 can modulate GREM1 expression in OSCC. GREM1 is an antagonist of bone morphogenetic protein, which plays a key role in multiple biological processes, including cancer biology.^10^ In particular, GREM1 plays an important role in certain malignancies by antagonizing bone morphogenetic proteins and regulating angiogenesis.^11^ In addition, Kim et al identified GREM1 as an OSCC‐related gene.^12^ Previous studies have suggested that GREM1 can inhibit BMP,^13^ which is a member of TGF‐β family^14^, ^15^, ^16^, ^17^ and have also suggested that interference with GREM1 expression may result in decreased TGF‐β expression.^18^ Insofar as GREM1 could potentially affect TGF‐β signal pathway, we conducted the present study with the main objective of exploring the effects of miR‐218 on OSCC and its mechanism involving GREM1 and the TGF‐β signalling pathway.

## 2 MATERIALS AND METHODS

### 2.1 Ethical statement

This study was conducted in strict accordance with the Helsinki declaration and approved by the medical ethics committee of Linyi People's Hospital (201708005). Participants provided written informed consent prior to the study. All animal experiments were conducted in accordance with the International Convention on the Ethics of Laboratory Animals and the relevant national regulations. All the animal experiments have been approved by the animal ethics committee of Linyi People's Hospital (201903012).

### 2.2 Gene expression omnibus (GEO) expression dataset retrieval and differential analysis

In the GEO database (https://www.ncbi.nlm.nih.gov/geo/), four expression data sets related to OSCC were obtained, namely GSE10121 (35 OSCC cases and 6 controls), GSE30784 (167 OSCC cases and 45 controls), GSE37991 (40 OSCC cases and 40 controls) and GSE74530 (6 OSCC cases). The R ‘limma’ package was used to analyse the difference between the OSCC group and the normal control group of the four expression data sets, and the differential gene expression dendrogram was constructed using the ‘pheatmap’ package. A Venn diagram of differential genes in the four expression data sets was constructed to determine the intersection of the differential genes.

### 2.3 Analysis of known gene retrieval and gene interaction in OSCC

DigSee (http://210.107.182.61/geneSearch/) is a text retrieval search engine to provide evidentiary sentences describing that ‘genes’ are involved in the development of ‘disease’ through ‘biological events’. The database was used to retrieve the known associated genes of OSCC, the first ten of which were selected for subsequent analysis. Using STRING database (https://string-db.org/), we analysed the interaction of the 10 genes and the above intersection genes in the expression data set. After using Cytoscape software (ver3.7.1) for visualization of the interaction, we constructed a gene interaction network diagram.

### 2.4 MiRNA prediction of GREM1 gene regulation

In the databases microRNA.org (http://34.236.212.39/microrna/home.do), miRDB (http://mirdb.org/miRDB/index.html), mirDIP (http://ophid.utoronto.ca/mirDIP/index.jsp#r), starBase (http://starbase.sysu.edu.cn/) and TargetScan (http://www.targetscan.org/vert_71/), we retrieved the regulatory miRNA of GREM1 and intersected the prediction results of the five databases. Afterwards, the binding site information of GREM1 and miRNA was obtained from the microRNA.org database.^19^

### 2.5 Study subjects

A total of 65 resected specimens (OSCC tissues and adjacent normal tissues) were collected from the patients with pathologically confirmed OSCC who underwent surgery resection at Linyi People's Hospital. The patients consisted of 43 males and 22 females (range 34‐78 years). The OSCC were categorized according to the American Joint Committee on Cancer tumour‐node‐metastasis (AJCC TNM) classification in 2010: stage I/II, n = 26; stage III, n = 39; high differentiation, n = 25; moderate and low differentiation, n = 40; liver metastases nodes, n = 29; non‐LNM, n = 36. All specimens were fixed with 10% formaldehyde, embedded with paraffin and cut into 8 μm‐thick sections for subsequent use.

### 2.6 Immunohistochemistry

The tissue sections were baked at 60°C for 1 hour, dewaxed with conventional xylene and hydrated with gradient ethanol. Next, the sections underwent incubation in phosphate‐buffered saline (PBS) containing 0.5% Triton‐X at room temperature for 20 minutes. After high‐pressure antigen retrieval for 2 minutes, the sections were heated at 95°C for 20 minutes with 0.01 M citrate buffer (pH = 6.0) and then immersed in 3% H_2_O_2_ for 15 minutes to block the exogenous peroxidase. Afterwards, the sections were sealed using sealing liquid conjugated with 3% bovine serum albumin (BSA) at 37ºC and gently shaken for 20‐30 minutes. The sections were then added with diluted primary antibodies rabbit polyclonal antibody GREM1 (1:1000, ab22138; Abcam, Cambridge, UK), and incubation was carried out at 37°C for 2 hours. Next, secondary antibody of goat anti‐rabbit conjugated with horseradish peroxidase (HRP) (1:1000 dilution; Zhongshan Bio‐tech CO., LTD, Guangdong, China) was added into sections, followed by incubation in the wet box for 30 minutes at 37°C. Subsequently, the sections were counterstained with haematoxylin at room temperature for 4 minutes, washed with running water to remove excess dye liquor and mounted with 10% glycerine/PBS. PBS was used to displace primary antibody as negative control (NC), and the known positive sections were used as positive controls. The sections were observed and photographed under a light microscope (XSP‐36; Bovision Optical Instrument Co., Ltd, Shenzhen, China) with selection of five randomly selected fields in each section, each containing 100 OSCC cells. The positive rate of GREM1 protein was quantitatively analysed by using Image‐Proplus image analysis software (Media Cybernetics, Silver Spring, MD, USA) under a high‐power microscope, and the mean optical density of GREM1‐positive staining was measured.^20^

### 2.7 Dual‐luciferase reporter gene assay

Human embryonic kidney HEK293T cells were cultured in Dulbecco's Modified Eagle Medium (DMEM) containing 10% foetal bovine serum (FBS) at 37°C with 5% CO_2_. The GREM1 3’‐untranslated region (UTR) fragment containing miR‐218 binding site was inserted into pmirGLO vector. GREM1 3’‐UTR fragment containing mutant of binding site was constructed using the point mutation method and inserted into pmirGLO vector. The insertion sequence was confirmed to be correct by sequencing. pmirGLO‐GREM1‐wild‐type (Wt) or pmirGLO‐GREM1‐mutant (Mut) recombinant vectors were co‐transfected with miR‐218 mimic or miR‐NC into HEK293T cells by liposome transfection and incubated for 48 hours. The firefly luciferase activity and renilla luciferase activity of HEK293T cells were detected successively based on the instructions in the dual‐luciferase reporter gene kit (E1910; Promega Company, Madison, WI, USA). Subsequently, the relative luciferase activity was regarded as the ratio of firefly luciferase activity and renilla luciferase activity. Specifically, for preparation of passive lysis buffer 1 × PLB: 5 × PLB was added to 4 × volumes of distilled water, mixed and stored at 4°C (<1 month). For preparation of LAR II: luciferase assay buffer II (10 mL in E1910 and E1960; 10 mL in E1980) was used to dissolve the lyophilized powder Luciferase Assay Substrate and was then stored at −20°C (<1 month) or −70°C (<1 year). For preparation of Stop & Glo^®^ reagent: (a) 2.1 mL 50 × Stop & Glo^®^ substrate was added to 10 mL Stop & Glo^®^ buffer, gently shaken in the brown Stop & Glo^®^ reagent bottle for 10 seconds and then stored at −20°C for 15 days; (b) For preparation of a small amount of Stop & Glo^®^ reagent: 50 × Stop & Glo^®^ substrate was added to Stop & Glo^®^ buffer to a final concentration of 1×. Then, 100 µL LAR Ⅱ was added to the detector tube, after which 20 µL PLB lysis solution was added and thoroughly mixed. The activity of firefly luciferase was detected, whereupon 100 µL Stop & Glo^®^ substrate was added to detect the activity of Renilla luciferase.

### 2.8 Cell transfection and grouping

Three human OSCC‐derived cell lines SAS, HSC4 (Human Science Research Resources Bank, Osaka, Japan) and CAL‐27 (American Type Culture Collection, ATCC, Manassas, VA, USA), and one normal human oral epithelial cell line HOEC (ATCC) were used in this study. CAL‐27, SAS and HSC4 were grown in DMEM (90% v/v) and foetal bovine serum (10% v/v), with the addition of penicillin (50 U/mL) and streptomycin (50 μg/mL). Cultured cells were maintained at 37°C under an atmosphere of 5% CO_2_. Upon reaching 70%‐80% confluence, the cells were sub‐cultured. The cells in a logarithmic growth phase were resuspended with DMEM culture solution containing 10% FBS with cell density adjusted to 3.5 × 10^4^ cells/mL and then seeded into a 96‐well plate overnight with 100 µL cell suspension fluid in each well. The following day, the cells were cultured in serum‐free DMEM culture fluid. After 24 hours, the cells were again cultured in DMEM culture solution containing 0.5% FBS. The cells were assigned into 6 groups: (a) the blank (untreated cells) group, the (b) NC group (cells transfected with miR‐218 NC sequence), (c) the miR‐218 mimic group (cells transfected with miR‐218 mimic sequence), (d) the miR‐218 inhibitor group (cells transfected with miR‐218 inhibitor sequence), (e) the si‐GREM1 group (cells transfected with si‐GREM1 sequence) and (f) the miR‐218 inhibitor + si‐GREM1 group (cells transfected with miR‐218 inhibitor and si‐GREM1 sequence). Cells were inoculated into 6‐well plates 24 hours before transfection. Once the cell density reached 30%‐50%, they were transfected based on the instructions of the Lipofectamine 2000 kit (11668‐019; Invitrogen, New York, CA, USA). The cells were cultured in cell culture wells at 37°C with 5% CO_2_ for 6‐8 hours. After subsequently culturing the cells in a complete culture medium for 24‐48 hours, the cells were collected, and their RNA and protein were extracted for analysis. The primer sequence for miR‐218 mimic was: 5′UUGUGCU UGAUCUAACCAUGU3′,^21^ and that for miR‐218 inhibitor was: 5′AACACGA ACUAGAUUGGUACA3′.

### 2.9 Reverse transcription quantitative polymerase chain reaction (RT‐qPCR)

Total RNA from tissues and cells was extracted by TRIzol (Invitrogen, Carlsbad, CA, USA) and a nanodrop2000 ultraviolet spectrophotometer (1011U; NanoDrop Technologies, Wilmington, DE, USA) was used to measure the absorbance value ratios (A_260_/A_230_ and A_260_/A_280_) and to confirm the concentration and purity of total RNA. Next, reverse transcription was conducted to obtain cDNA according to the instructions of TaqMan MicroRNA Assays Reverse Transcription primer (4427975; Applied Biosystems Inc, Carlsbad, CA, USA). The primers of GREM1, TGF‐β1, Smad4, p21, E‐cadherin, Vimentin and Snai were designed and then synthesized by TaKaRa company (Kusatsu, Japan) (Table 1). ABI7500 quantitative PCR instrument (7500; Applied Biosystems Inc) was applied to conduct RT‐qPCR detection. The ratio of target gene expression between the experiment group and the control group was determined using the 2^−ΔΔCt^ method, with β‐actin used as the internal control.

**TABLE 1:** The primer sequences for reverse transcription quantitative polymerase chain reaction

Gene	Primer sequence
GREM1	F: 5′‐TGCAACAGTCGCACCATCAT‐3′
	R: 5′‐TGCAGAAGGAGCAGGACTGA‐3′
TGF‐β1	F: 5′‐CAGATCCTGTCCAAGCTA‐3′
	R: 5′‐CCTTGGCGTAGTAGTCG‐3′
Smad4	F: 5′‐CTCTAAACCTCAGGCCACATC‐3′
	R: 5′‐CAATACCTCCTCCATCAAAGC‐3′
p21	F: 5′‐TGTCCGCGAGGATGCGTGTTC‐3′
	R: 5′‐TTCTGACATGGCGCCTGCCGC‐3′
E‐cadherin	F: 5′‐GGAGCAGAAAGCAGAACCC‐3′
	R: 5′‐TTCCTTCCACGAAACCAGTG‐3′
Vimentin	F: 5′‐CTCTCAAAGATGCCCAGGAG‐3′
	R: 5′‐GCACGATCCAACTCTTCCTC‐3′
Snail	F: 5′‐TTCTTCGCTACTGCTGCG‐3′
	R: 5′‐GGGCAGGTATGGAGAGGAAGA‐3′
β‐actin	F: 5′‐GAGCCTCGCCTTTGCCGATCC‐3′
	R: 5′‐CGATGCCGTGCTCGATGGGG‐3′
miR‐218	F: 5′‐CGAGTGCATTTGTGCTTGATCTA‐3′
	R: 5′‐TGGTGTCGTGGAGTCG‐3′

Abbreviations: F, forward; GREM1, Gremlin1; miR‐218, microRNA‐218; R, reverse.

### 2.10 Western blot analysis

The total protein of cells to be detected was extracted using Radio Immunoprecipitation Assay (RIPA) cell lysis buffer (BB‐3209; BestBio Co., Ltd., Shanghai, China), followed by electrophoretic separation with sodium dodecyl sulphate polyacrylamide gel electropheresis (SDS‐PAGE) and transferred to polyvinylidene fluoride (PVDF) membrane. The membrane was blocked with solution for 1 hour and added with primary antibody of rabbit anti‐human clone antibody for incubation at 4°C overnight: GREM1 (1:1000, ab140010), TGF‐β1 (1:200, ab92486), Smad4 (1:1000, ab40759), p21 (1:1000, ab109520), E‐cadherin (1:10 000, ab40772), Vimentin (1:1000, ab92547), Snail (1:500, ab53519) and GAPDH (1:10 000, ab181602) (all the antibodies were purchased from Abcam, Cambridge, UK), followed by incubation with gentle shaking for 1 hour at 37°C along with IgG goat anti‐rabbit secondary antibody. Then, the membrane was developed, and the relative expression of target proteins calculated as the ratio of the grey value of the target protein band to the grey value of internal control band in the same sample, where GAPDH served as internal control. The relative expression of proteins was calculated as the staining intensity of the target protein/the corresponding GAPDH staining intensity. Each experiment was conducted in triplicate, with the average value calculated.

### 2.11 Transwell assay

The apical chamber of the Transwell chambers was coated with Matrigel diluted in pre‐cooled serum‐free DMEM medium and incubated in an incubator at 37°C for 4‐5 hours. After the solidification of Matrigel, 100 μL serum‐free medium was used to dilute the transfected cells to make a suspension containing 1 × 10^6^ cells/mL, followed by inoculation. The basolateral chamber was added with 500 μL DMEM containing 20% FBS. Each group was set three duplicate wells. After incubation at 37°C for 24 hours with 5% CO_2_, the Transwell chamber was fixed with 5% glutaraldehyde at 4°C and then stained with 0.1% crystal violet for 5 minutes. After removal of the surface adhering cells with cotton balls, the cells were observed with an inverted fluorescence microscope (Nikon TE2000, Tokyo, Japan). The mean number of cells in three visual fields was measured.

### 2.12 Scratch test

The transfected cells were incubated in an incubator at 37°C for 24 hours with 5% CO_2_, whereupon the monolayer cells were wounded by scratching with a sterile 10 μL pipette tip. Next, the cells were added with serum‐free medium and incubated for 24 hours. Cell migration at 0 and 24 hours was observed using the inverted microscope. Three sites were selected in each group for photography and measurement of the relative migration distance between cells on either side of the scratch. The distance difference was divided by two to obtain relative migration distance, and cell migration rate was calculated as the relative migration distance/the distance from the scratch margin to centerline at 0 hours. The experiment was conducted in triplicate.

### 2.13 3‐(4,5‐dimethyl thiazol‐2‐yl)‐2,5‐diphenyl tetrazolium bromide (MTT) assay

Tissues and cells were selected for the experiment. Cell suspensions were placed in a centrifuge tube and triturated using a sterile straw. Then, the single cell suspension was stained by trypan blue to count the numbers of living cells. The cell suspensions were seeded into a 96‐well plate with 180 µL containing 1 × 10^4^ cells/well and then incubated at 37°C with 5% CO_2_ for 16‐18 hours. Next, each well was added with 20 µL 5% MTT solution in subdued light, and then shaken and mixed. Following this, the 96‐well plate was further incubated at 37°C with 5% CO_2_ in the dark for 4 hours. Afterwards, 100 µL dimethyl sulfoxide (DMSO) was added and the plate was shaken for 10 minutes to aid the full dissolution of crystals. After standing for 15 minutes, an enzyme‐linked immunosorbent assay (SAF‐680T, Multiskan GO, Thermo, Boston, USA) was used to measure the optical density (OD) of the wells. A cell growth curve was drawn with the MTT processing time as the abscissa and the OD value as ordinate axis.

### 2.14 Xenograft tumour in nude mice

A subcutaneous xenograft tumour was established as follows: 36 athymic female nude mice (4‐6 weeks old and 20‐28 g) were raised in housing at constant temperature (25‐27°C) and humidity of 40%‐50%. Stable knockdown of GREM1 cells was constructed by lentivirus infection. When cell confluence after transfection reached 80%‐90%, cells were digested and centrifuged. Cells were resuspended and counted, with adjustment of the cell concentration to 1 × 10^7^ cells/mL. Then, 20 μL cell suspension was injected into axillary subcutaneous of each of six nude mice in each group. miR‐218 agomir, miR‐218 antagomir, and the corresponding NC were injected into the mice via a tail vein to induce overexpression or knockdown of miR‐218 in vivo. The mice were killed with CO_2_ after 6 weeks, and the xenograft tumours were extracted and measured with Vernier calipers. The size of tumours was calculated as (a × b^2^)/2, where a represents the maximum diameter of tumour and b the minimum diameter.

### 2.15 Liver metastasis in nude mice

Cells were treated as described above, and lentivirus expressing shRNAs targeting human GREM1 transcripts was generated using the Mission PLKO.1 lentivirus system from Sigma (St. Louis, MO, USA). A lentivirus expressing a non‐targeting scrambled shRNA was used as a normal control. CAL‐27 cells were transduced with lentivirus as per the manufacturer's instructions, and stable cell lines were generated by selection with puromycin. Then, cell suspension was injected into the nude mice (n = 36) via a tail vein to induce metastasis, with six mice in each treatment group. Cal‐27 cells were injected into the tail veins along with the miR‐218 mimic or miR‐218 inhibitor every 7 days for 1 month. After 30 days, the mice were killed and the lungs were excised and photographed, and tumour nodes visible on the lung surface were counted.^22^

### 2.16 Statistical analysis

Statistical analysis was conducted using SPSS19.0 (IBM Co., Armonk, NY, USA). The results are expressed as the mean ± standard deviation. Cell experiments were independently repeated at least three times. If data obeyed normal distribution and homogeneity of variance, data between two groups with paired design were compared by paired *t* test, while data between two groups with unpaired design were compared by unpaired *t* test. Data among multiple groups were compared by one‐way analysis of variance (ANOVA), followed by Tukey's post hoc test. Data at different time points among multiple groups were compared by repeated measurement ANOVA, followed by Bonferroni post hoc test. *P* < 0.05 was considered statistically significant.

## 3 RESULTS

### 3.1 In OSCC, miR‐218 may play an important role in the TGF‐β signalling pathway through binding to GREM1 mRNA

**FIGURE 1:**
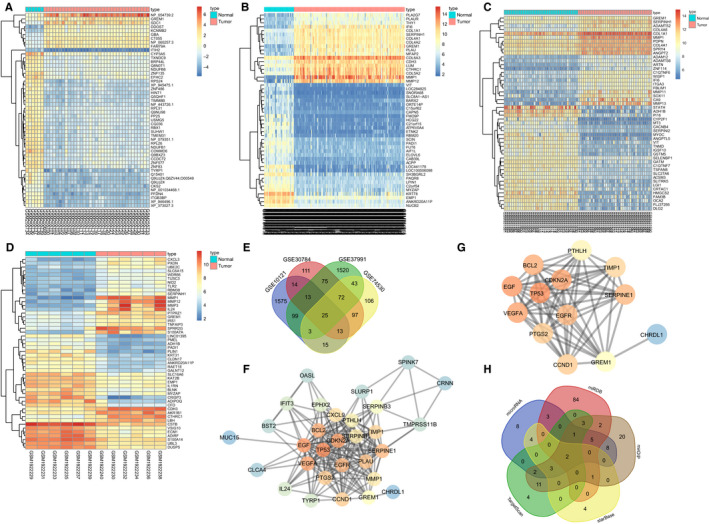
Bioinformatics prediction of the role of miR‐218‐GREM1 axis in the development of OSCC. A, Expression heat map of differentially expressed genes in OSCC‐related data set GSE10121. B, Expression heat map of differentially expressed genes in OSCC‐related data set GSE30784. C, Expression heat map of differentially expressed genes in OSCC‐related data set GSE37791. D, Expression heat map of differentially expressed genes in OSCC‐related data set GSE74530. The abscissa represents the sample numbers, and the ordinate represents the differentially expressed genes. The left dendrogram indicates the clustering of gene expression. The histogram in the upper right is the colour scale, and each rectangle in the figure corresponds to a sample expression value. E, Venn map of four differentially expressed genes of OSCC expression dataset. Four different colours of ellipse represent the differentially expressed genes of four expression data sets, respectively. Cross regions are represented by the intersection of four expression data sets, where the middle part indicates the intersection of four expression data sets. F, Microarray analysis obtained a network of known genes interacted with the genes retrieved from the database. Each circle represents a gene, and the circle colour indicates the core position of the gene along the network. The higher the core degree is, the brighter the colour would be. G, Gene interacting with GREM1 predicted to the network. H, The prediction results of the miRNA regulating GREM1 gene. The different colours represent the prediction results of different databases. The cross‐section indicates the intersection of the prediction results of each database, and the middle part indicates the intersection of the results of the five databases

Using GEO database, 4 OSCC expression data sets, GSE10121 (35 OSCC cases and 6 controls), GSE30784 (167 OSCC cases and 45 controls), GSE37791 (40 OSCC cases and 40 controls) and GSE74530 (6 OSCC cases), were retrieved. A total of 1757, 420, 1850 and 374 differentially expressed genes were obtained from differential expression analysis of genes in normal control group and OSCC group in the 4 expression data sets (Figure 1A‐D). Meanwhile, the expression dendrogram of 50 significant differentially expressed genes in these 4 expression data sets were constructed (Figure 1A‐D). To further screen the genes associated with OSCC, Venn analysis of the differentially expressed genes from the 4 expression data sets was performed to obtain intersections (Figure 1E). The results revealed 25 genes at the intersection from all 4 expression data sets, which were selected as candidate genes. DigSee database was used to retrieve the known genes associated with OSCC, and the top 10 were selected for subsequent analysis (Table 2). We analysed the genetic interactions between the 10 known retrieved genes and the 25 candidate genes obtained from the expression data set (Figure 1F). The results showed that, among the 25 candidate differentially expressed genes, GREM1 gene was in the most core position and had an interaction with the ten known genes associated with OSCC (Figure 1G), suggesting that GREM1 may play an important role in the development of OSCC. In addition, the expression of GREM1 gene in the OSCC samples was significantly up‐regulated according to the dendrogram of the differential gene expression in the 4 expression datasets. The bibliographic retrieval of GREM1 gene‐related functions showed that GREM1 can affect the development of cancer and other diseases through the TGF‐β signalling pathway,^23^ which has elsewhere been reported to be involved in the development of OSCC.^24^, ^25^ These results, along with previous literature, suggest that the GREM1 gene is highly likely to participate in the development of OSCC, doing so via the TGF‐beta signalling pathway.

**TABLE 2:** The details of oral squamous cell carcinoma‐related genes by DigSee database retrieval

Searched genes	Gene name	Number of abstracts
TP53	Tumour protein p53	671
EGFR	Epidermal growth factor receptor	296
CDKN2A	Cyclin‐dependent kinase inhibitor 2A	201
SERPINB3	Serpin family B member 3	185
VEGFA	Vascular endothelial growth factor A	153
BCL2	B‐cell lymphoma 2	151
EGF	Epidermal growth factor	142
CCND1	Cyclin D1	138
CDKN1A	Cyclin‐dependent kinase inhibitor 1A	132
PTGS2	Prostaglandin‐endoperoxide synthase 2	130

To elaborate further the mechanism of GREM1 gene in OSCC, 5 databases were used to predict miRNAs mediating GREM1 and the intersections of the predicted results were determined (Figure 1H). This showed that there were 2 miRNAs at the intersection of the five databases, namely miR‐218 and miR‐128. A further literature search revealed some reports that miR‐218 expression was related to the radiation sensitivity of OSCC.^9^, ^26^ However, the regulatory targets and mechanisms of miR‐218 in OSCC have not previously been reported. The above analysis and relevant literature show that miR‐218 is highly likely to inhibit GREM1 gene expression through the TGF‐β signalling pathway, thus affecting the development of OSCC.

### 3.2 Up‐regulated GREM1 and down‐regulated miR‐218 are found in OSCC

**FIGURE 2:**
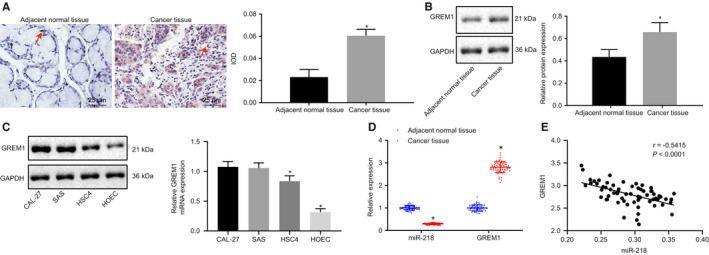
miR‐218 is poorly expressed while GREM1 is highly expressed in OSCC tissues. A, GREM1‐positive cells and relative statistics in OSCC tissues and adjacent normal tissues based on immunohistochemistry (×400). B, Expression of GREM1 and relative statistics in OSCC tissues and adjacent normal tissues according to Western blot. C, Protein and mRNA expression of GREM1 in CAL‐27, SAS and HSC4 cells. D, GREM1 and miR‐218 expression in OSCC tissues and adjacent normal tissues by RT‐qPCR. n = 65. *P < 0.05 compared with adjacent normal tissues. (Data in panel B were enumeration data analysed using chi‐square test, while data in panel C were measurement data expressed as the mean ± standard deviation). Paired *t* test was conducted concerning raw data of a single indicator between two groups. E, Pearson correlation analysis of relation between expression of GREM1 and expression of miR‐218. n = 65

Immunohistochemistry showed that the positive staining of GREM1 had a brown yellow appearance. Immunohistochemistry and Western blot analyses showed that GREM1 protein expression was much higher in OSCC tissues when compared to adjacent normal tissues (*P* < 0.02, Figure 2A,B). At the same time, we detected the protein and mRNA expression of GREM1 in CAL‐27, SAS and HSC4 cells, finding that the expression of GREM1 was relatively higher in CAL‐27 and SAS cells. Thus, we also verified the action of miR‐218.targeted regulation of GREM1 in SAS cell lines (Figure 2C). RT‐qPCR was used to detect relative expression of miR‐218 and GREM1 in OSCC tissues and adjacent normal tissues. The results revealed a low expression of miR‐218 in OSCC tissues compared with that in adjacent normal tissues, while the expression of GREM1 was high in the tumour tissues (Figure 2D; n = 65). We further analysed the correlation between miR‐218 and GREM1 expression, finding that miR‐218 was negatively correlated with GREM1 expression (*P* < 0.05) (Figure 2E). In addition, the analysis of the relationship between clinicopathological features of patients with OSCC and the expression of miR‐218 and GREM1 showed that the expressions of miR‐218 and GREM1 were closely related to LNM and TNM stage (both *P* < 0.05) but had no relationship with patient age or gender, or the size of OSCC tumour and degree of differentiation (Table 3). These findings suggest that OSCC tissues had increased expression of GREM1 and decreased miR‐218 expression.

**TABLE 3:** The relationship between miR‐218 and GREM1 expression and clinicopathological features of in OSCC

Clinicopathological features	Case	miR‐218 expression	*P*	GREM1 expression	*P*
Age (y)					
<54	38	0.295 ± 0.034	.446	2.766 ± 0.212	.104
≥54	27	0.288 ± 0.039		2.865 ± 0.272	
Gender					
Male	43	0.291 ± 0.038	.677	2.802 ± 0.251	.815
Female	22	0.295 ± 0.033		2.817 ± 0.229	
Tumour size (cm)					
<4	47	0.296 ± 0.035	.133	2.807 ± 0.247	.988
≥4	18	0.281 ± 0.037		2.808 ± 0.236	
TNM stage					
Ӏ, II	26	0.317 ± 0.024	.001	2.618 ± 0.168	.001
III	39	0.275 ± 0.033		2.933 ± 0.198	
Lymph node metastasis					
No	36	0.317 ± 0.023	.001	2.698 ± 0.198	.001
Yes	29	0.261 ± 0.023		2.943 ± 0.224	
Pathological grade					
High differentiation	25	0.288 ± 0.033	.451	2.775 ± 0.218	.404
Medium and low differentiation	40	0.295 ± 0.038		2.827 ± 0.257	

Abbreviations: GREM1, Gremlin1; miR‐218, microRNA‐218; OSCC, oral squamous cell carcinoma; TNM, tumour node metastasis.

### 3.3 GREM1 may be target gene of miR‐218

**FIGURE 3:**
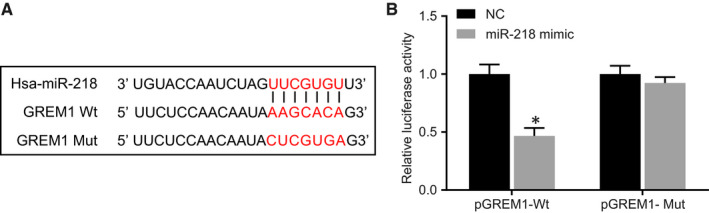
miR‐218 targets GREM1 in OSCC cells. A, Biology prediction website microRNA.org shows miR‐218 binds to GREM1 [MirSVR: the lower the score of thermodynamic stability (≤−0.1), the stronger the binding stability of miRNA‐mRNA, the greater the possibility of corresponding miRNA down‐regulating relative genes; PhastCos: the strength of evolutionary conservatism of non‐translation regions of genes in various species (≥0), and the more conservative the PhastCos, the better].^41^ B, Luciferase activity of pGREM1‐wt and pGREM1‐mut in cell treated with miR‐218 mimic or NC by dual‐luciferase reporter assay. The measurement data were expressed as the mean ± standard deviation and analysed by unpaired *t* test. The cell experiment was independently repeated at least three times. *P < 0.05 compared with the NC group

According to the bioinformatics online prediction tool microRNA.org, GREM1 might be a potential target gene of miR‐218 (Figure 3A). Dual‐luciferase report gene assay (Figure 3B) was used to confirm the targeting relationship between miR‐218 and GREM1, showing that, compared with that in the NC group, the luciferase activity of pGREM1‐Wt was markedly decreased in the miR‐218 mimic group (*P* < 0.05), while the luciferase activity of pGREM1‐Mut did not differ between the two groups (*P* > 0.05). Therefore, GREM1 was the potential target gene of miR‐218, which could negatively regulate its expression.

### 3.4 Overexpressed miR‐218 may inhibit the EMT progression via the TGF‐β signalling pathway

**FIGURE 4:**
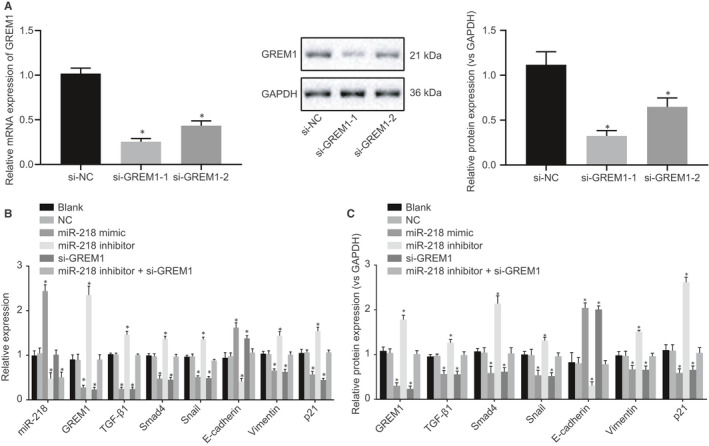
Up‐regulated miR‐218 may inhibit EMT progression in CAL‐27 cells through the TGF‐β signalling pathway. A, The knockdown efficiency of GREM1 determined by RT‐qPCR and Western blot analysis. B, miR‐218 expression and mRNA expressions of Smad4, p21, E‐cadherin, GREM1, TGF‐β1, Vimentin and Snail in OSCC cells with treatment of up‐regulation of miR‐218 and down‐regulation of GREM1 by RT‐qPCR (after 48 h of transfection). C, Smad4, p21, E‐cadherin, GREM1, TGF‐β1, Vimentin and Snail protein expression in OSCC cells with treatment of up‐regulation of miR‐218 and down‐regulation of GREM1 by Western blot analysis (after 72 h of transfection). The measurement data were analysed by one‐way ANOVA, followed by Tukey's post hoc test. The cell experiment was independently repeated at least 3 times. *P < 0.05 compared with the blank group and the NC group

To verify further the impact of GREM1 knockdown on OSCC behaviour, we tested the knockdown efficiency of GREM1, finding relatively higher knockdown efficiency of si‐GREM1‐1 (Figure 4A), which was used for subsequent studies. We then conducted RT‐qPCR and Western blot analysis to evaluate the expressions of GREM1, TGF‐β1, Smad4, p21, E‐cadherin, Vimentin and Snail in OSCC cells CAL‐27 (Figure 4B,C) and SAS (Figure S1A‐C). The results showed no significant difference in the expression of miR‐218, GREM1, TGF‐β1, Smad4, p21, Vimentin, E‐cadherin and Snail between the blank group and the NC group (all *P* > 0.05). Compared with the blank and NC groups, miR‐218 expression, E‐cadherin mRNA and protein expression were significantly higher in the miR‐218 mimic group, while GREM1, TGF‐β1, Smad4, p21, Vimentin and Snail mRNA and protein expression were all lower (all *P* < 0.05), which was opposite in the miR‐218 inhibitor group; in the si‐GREM1 group, E‐cadherin mRNA and protein expression was enhanced, and TGF‐β1, Smad4, p21, Vimentin and Snail mRNA and protein expression was decreased, while the opposite changes were observed in the miR‐218 inhibitor + si‐GREM1 group (all *P* < 0.05). These findings showed that the overexpression of miR‐218 or silencing of GREM1 could result in the suppression of the EMT progression via the TGF‐β signalling pathway.

### 3.5 Up‐regulated miR‐218 or silencing GREM1 suppresses the OSCC cell invasion, migration and proliferation

**FIGURE 5:**
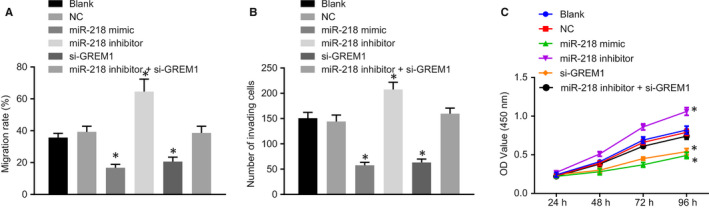
Overexpressed miR‐218 or silenced GREM1 suppresses the invasion, migration and proliferation of CAL‐27 cells. A, Cell migration in OSCC with the overexpression of miR‐218 and down‐regulation of GREM1 detected by scratch test. B, Transwell assay of cell invasion in OSCC with enforced miR‐218 expression and reduced GREM1 expression. C, Cell viability in OSCC with up‐regulated miR‐218 and down‐regulated GREM1 based on the results of MTT assay. The measurement data in panel C and D were analysed by one‐way ANOVA, followed by Tukey's post hoc test. The measurement data in panel C and D were analysed by repeated measurement ANOVA, followed by Bonferroni post hoc test. The cell experiment was independently repeated at least 3 times. *P < 0.05 compared with the blank group and the NC group

Transwell assay and scratch test were conducted to elucidate the effects of miR‐218 on invasion and migration in OSCC cells CAL‐27 (Figure 5; Figure S3) and SAS (Figure S2). The results showed that, in comparison with the blank group and the NC group, the miR‐218 mimic and the si‐GREM1 groups showed a significant decline in cell invasion and migration (all *P* < 0.05), while the miR‐218 inhibitor group showed elevated cell invasion and migration (*P* < 0.05), thus revealing that miR‐218 up‐regulation remarkably inhibited cell invasion and migration in OSCC cells. There were no notable differences in cell invasion and migration among the miR‐218 inhibitor + si‐GREM1 group, the blank group and the NC group (Figure 5A,B; Figures S2A‐D and S3A,B). Meanwhile, the MTT assay was performed to investigate further the viability of transfected OSCC cells. The results showed that, compared with the blank and NC groups, cell viability was prominently elevated in the miR‐218 inhibitor group, but was markedly reduced in the miR‐218 mimic and si‐GREM1 groups (all *P* < 0.05). There were no prominent differences in the miR‐218 inhibitor + si‐GREM1 group, the blank group and the NC group (*P* > 0.05; Figure 5C; Figure S2E). The aforementioned findings imply that overexpressed miR‐218 and silenced GREM1 could suppress the invasion, migration and proliferation in OSCC cells.

### 3.6 Overexpressed miR‐218 inhibits tumour growth and liver metastasis in nude mice with OSCC

**FIGURE 6:**
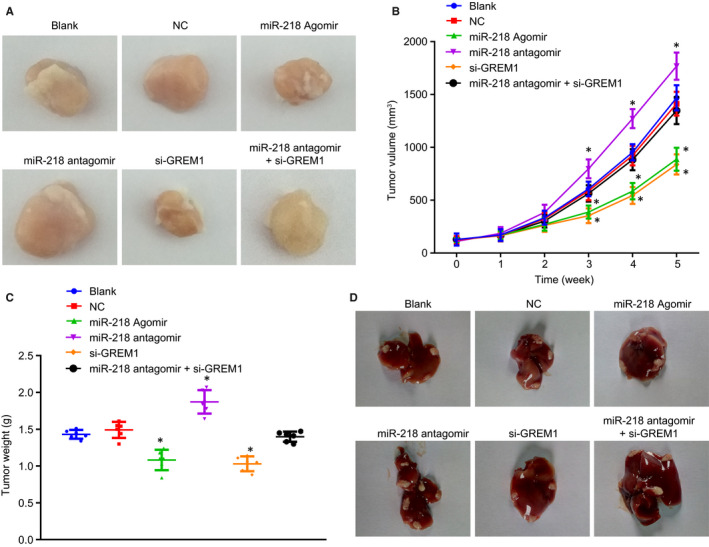
Overexpressed miR‐218 inhibits tumour growth and liver metastasis of nude mice with OSCC. A, Observation of tumours in each group. B, Tumour volume in each group. C, Tumour weight in each group. D, Observation of the number of liver metastases nodes of OSCC cells. The measurement data in panel C were analysed by one‐way ANOVA, followed by Tukey's post hoc test. The measurement data in panel B were analysed by repeated measurement ANOVA, followed by Bonferroni post hoc test. n = 6. *P < 0.05 compared with the blank group and the NC group

The effect of miR‐218 on tumour growth and liver metastasis in OSCC cells was verified in vivo by injecting nude mice with stably transfected OSCC cells constructed with lentivirus. The results (Figure 6A‐C) suggested that, compared with the blank and NC groups, the final tumour weight and the growth rate in mice in the miR‐218 agomir and si‐GREM1 groups was lower, while growth rate and final volume were higher in the miR‐218 antagomir group (all *P* < 0.05). There was no evident difference in these tumour results among the miR‐218 antagomir + si‐GREM1, blank and NC groups (all *P* > 0.05). The number of liver metastasis nodes in the miR‐218 agomir group and the si‐GREM1 group was lower compared with the blank group, while the number of liver metastasis nodes in the miR‐218 antagomir group was higher than that in the blank and NC groups (all *P* < 0.05). Furthermore, the number of liver metastasis nodes did not differ among the miR‐218 antagomir + si‐GREM1, blank and NC groups (all *P* > 0.05; Figure 6D). The findings show that overexpressed miR‐218 or silencing GREM1 led to the inhibition of tumour growth and liver metastasis of nude mice with OSCC.

## 4 DISCUSSION

OSCC is the most prevalent malignancy of the head and neck region, and its occurrence rate has increased in recent years.^27^ However, current treatment modalities for OSCC, which include extensive surgery, radiotherapy, chemotherapy or concurrent chemo‐radiation, have low efficacy in patients with advanced OSCC, due to the increased occurrence of tumour recurrence and metastasis.^9^ Therefore, identifying the underlying factors involved in the recurrence of OSCC is pivotal to reduce its postoperative recurrence and obtained better survival.^28^ A previous study has demonstrated that miR‐218 could serve as a potential cancer suppressor in human cancers.^29^ Thus, our study investigated the effect of miR‐218 on the regulation of cell invasion and migration in OSCC cells. Our findings demonstrated that miR‐218 might inactivate the TGF‐β signalling pathway by targeting GREM1, thereby inhibiting OSCC cell proliferation, migration, invasion, EMT and liver metastasis in OSCC cells.

In the initial experiment, we found that OSCC tissues showed down‐regulated expression of miR‐218 and up‐regulated expression of GREM1 compared with adjacent normal tissues. In the human genome, the miR‐218 precursor genes consist of miR‐218‐1 and miR‐218‐2, which reside in the introns of SLIT2 and SLIT3 genes. The promoter hypermethylation of SLIT2 and SLIT3 genes results in the down‐regulation of miR‐218 expression in cancer cells.^8^ Uesugi et al previously suggested that miR‐218 and miR‐585 expression is decreased in OSCC through DNA hypermethylation,^30^ which is consistent with our present findings. Moreover, Li et al demonstrated that hepatocellular carcinoma tissues had reduced levels of miR‐218 expression, which conceivably inhibits cell proliferation and promotes cell apoptosis,^31^ thus indicating that miR‐218 can inhibit cancer occurrence and progression mainly by suppressing cancer cell proliferation and invasion through the targeting of cancer genes.^32^ Another study demonstrated that reduced miR‐218 expression in gastric cancer was associated with advanced clinical stage, LNM and absence of accurate prognosis, while the expression of miR‐218 in metastatic cells had the ability to suppress migration, invasion and metastasis formation in vitro and in vivo.^33^ As a member of the DAN family of bone morphogenetic protein (BMP) antagonists, GREM1 is involved in the regulation of numerous cell functions in developing and adult tissues.^34^ Interestingly, cancer‐associated fibroblasts (CAFs) of human basal cell carcinomas have high levels of GREM1 expression, which promote the proliferation of cultured BCC cells.^35^ Based on the biology prediction website microRNA.org and present results of the dual‐luciferase reporter gene assay, GREM1 was identified as the target gene of miR‐218, which could negatively regulate the expression of GREM1.

Our study also demonstrated that the overexpression of miR‐218 or silencing GREM1 could suppress the invasion, migration and proliferation of OSCC cells by inhibiting the expressions of TGF‐β, Vimentin and Snail, while promoting the expressions of Smad, p21 and E‐cadherin. Tatarano et al similarly observed that miR‐218 expression could influence down‐regulated oncogenic genes and up‐regulated tumour suppressive genes.^36^ GREM1 plays a suppressive role by binding to BMP dimers, thus preventing their interaction with BMP receptors, as well as inhibiting BMP secretion and improving extracellular BMP endocytosis. We note that BMPs are members of the TGF‐β super‐family.^37^ As a multifunctional cytokine, TGF‐β1 can regulate a complicated signalling and inflammatory network and has dual effects in tumour development.^38^ Moreover, networks regulated by Smad‐independent TGF‐β also can be found in the well‐characterized Smad signalling pathway. The activation of signalling pathways leads to the activation of transcriptional regulators such as Snail, which is a crucial regulator of EMT, and can suppress the gene expression of E‐cadherin, which is a key feature of the epithelial phenotype.^39^ Through tumour formation in nude mice, we also found that the overexpression of miR‐218 resulted in the inhibition of tumour growth and liver metastasis. Tian et al reported that overexpression of miR‐218 in oesophageal squamous cell carcinoma cell lines suppressed cell proliferation, colony formation, migration and invasion and also inhibited tumour growth in nude mice.^40^

## 5 CONCLUSION

**FIGURE 7:**
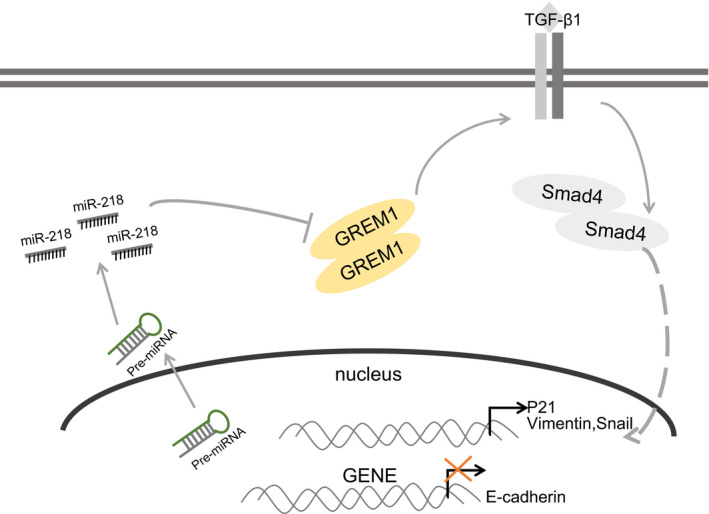
miR‐218 could inhibit OSCC proliferation, migration, invasion, EMT and liver metastasis through the TGF‐β signalling pathway by regulating GREM1. Thus, miR‐218 can serve as a cancer suppressor in OSCC and a potential predictor for the prognosis of OSCC

The key finding from this study is our demonstration that miR‐218 could inhibit OSCC proliferation, migration, invasion, EMT and liver metastasis by inactivating the TGF‐β signalling pathway via GREM1 down‐regulation. Thus, miR‐218 can serve as a cancer suppressor in OSCC and has potential as a predictor for the prognosis of OSCC (Figure 7). These findings may present this pathway as a promising target for development of novel OSCC therapies. However, the specific underlying mechanism of miR‐218 in OSCC shall require further elucidation in larger surgical specimens and in alternate cell lines.

## CONFLICT OF INTERESTS

The authors declare that they have no competing interests.

## AUTHOR CONTRIBUTION

**Yanpeng Wang:** Conceptualization (lead); data curation (equal); formal analysis (equal); investigation (lead); methodology (lead); writing – original draft (lead); writing – review and editing (equal). **Yifeng Jiang:** Data curation (equal); formal analysis (equal); methodology (supporting); supervision (lead); validation (lead); visualization (equal); writing – original draft (supporting); writing – review and editing (equal). **Long Chen:** Data curation (equal); formal analysis (equal); methodology (supporting); resources (equal); software (equal); visualization (equal); writing – review and editing (equal).

## Supporting information

Fig S1

Fig S2

Fig S3
