# Supplementary figures and images for: Role of miR‐218‐GREM1 axis in epithelial‐mesenchymal transition of oral squamous cell carcinoma: An in vivo and vitro study based on microarray data
Source: J Cell Mol Med. 2020 Oct 27;24(23):13824–36. doi: 10.1111/jcmm.15972 (PMC7754042; doi:10.1111/jcmm.15972)

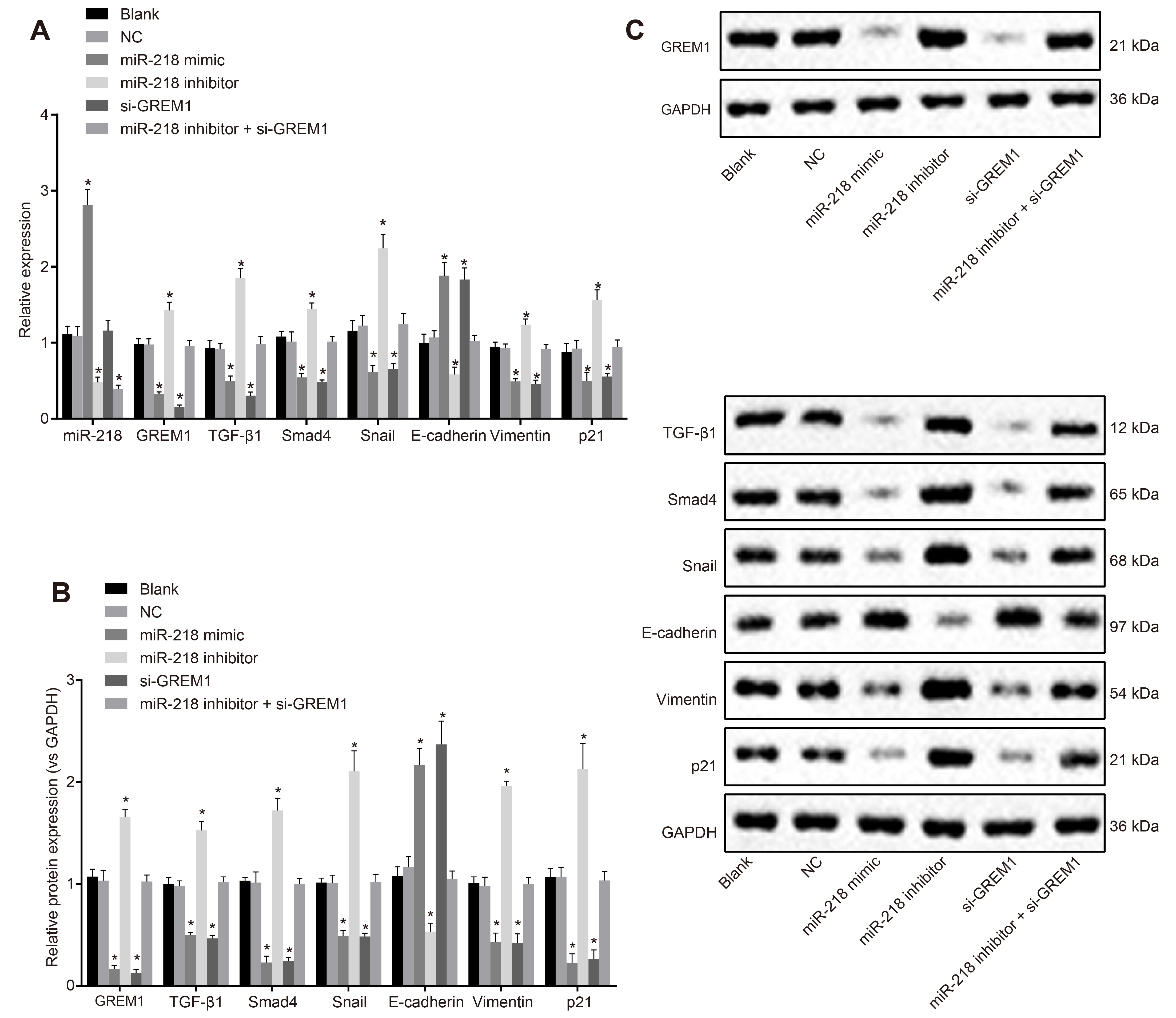

Supplement: Supplementary file 1 — Fig S1 [file JCMM-24-13824-s001.jpg]

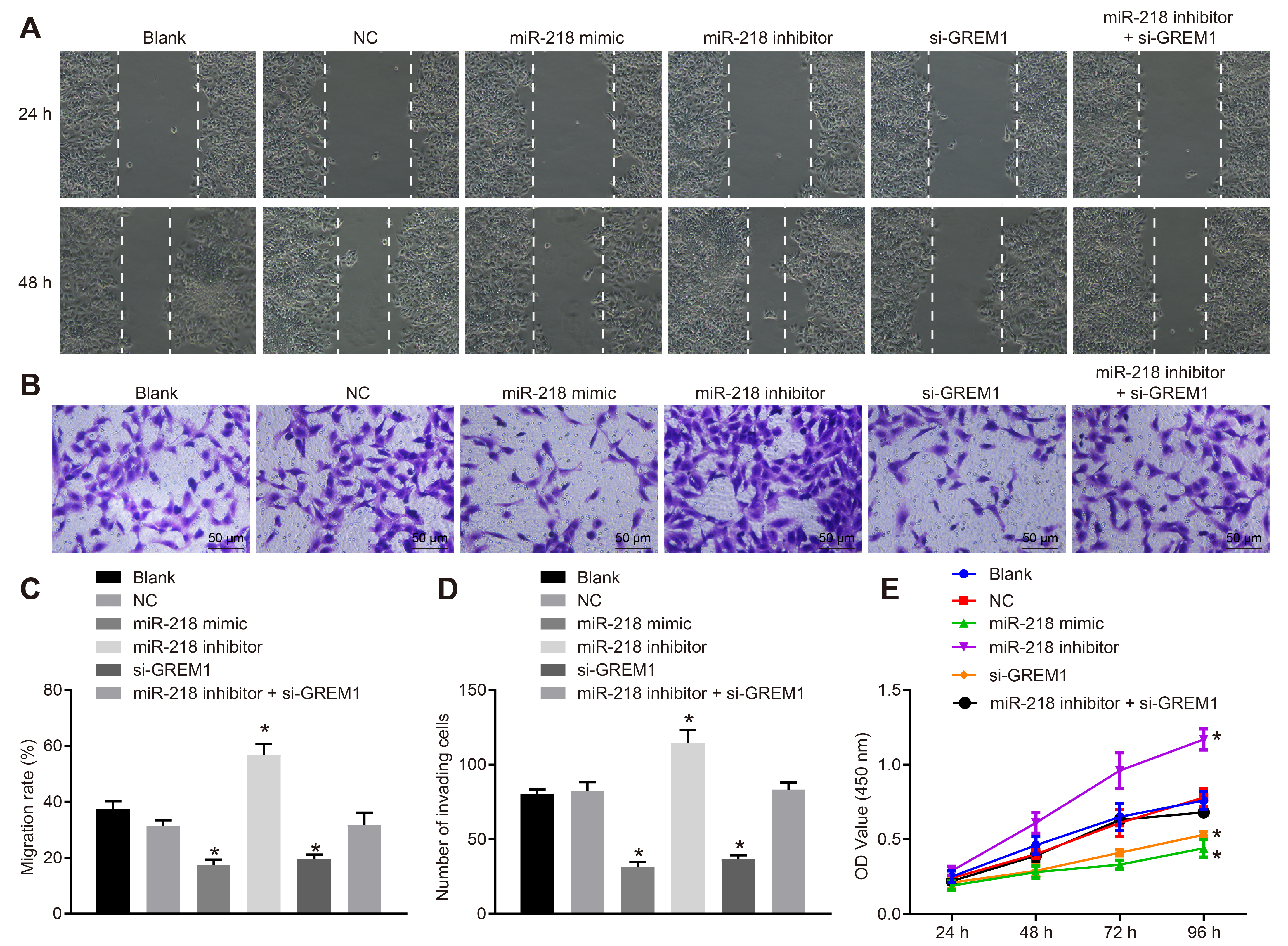

Supplement: Supplementary file 2 — Fig S2 [file JCMM-24-13824-s002.jpg]

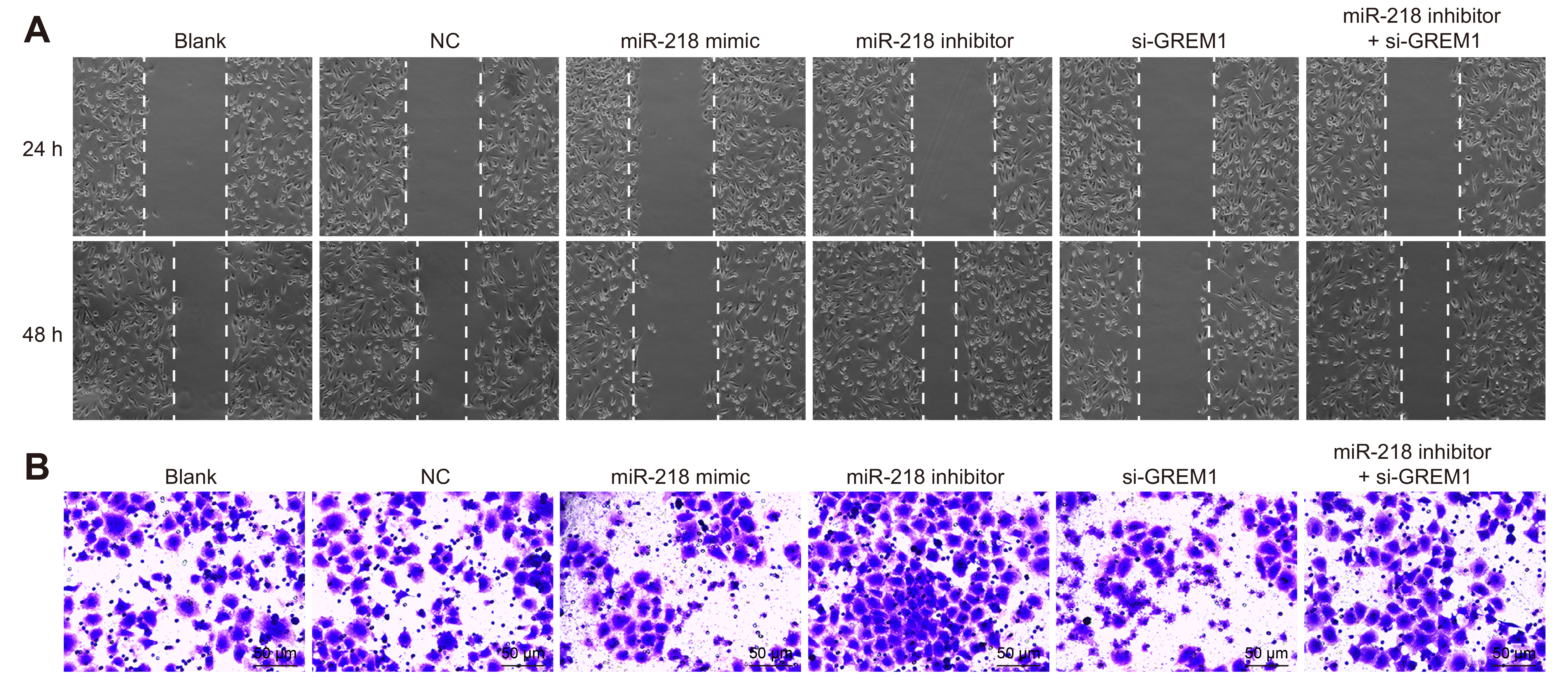

Supplement: Supplementary file 3 — Fig S3 [file JCMM-24-13824-s003.jpg]
